# Physical Exercise and Mental Health: The Routes of a Reciprocal Relation

**DOI:** 10.3390/ijerph182312364

**Published:** 2021-11-24

**Authors:** Chiara Fossati, Guglielmo Torre, Sebastiano Vasta, Arrigo Giombini, Federico Quaranta, Rocco Papalia, Fabio Pigozzi

**Affiliations:** 1 Department of Movement, Human and Health Sciences, University of Rome “Foro Italico”, 00135 Rome, Italy; chiara.fossati@uniroma4.it (C.F.); arrigo.giombini@uniroma4.it (A.G.); federico.quaranta@uniroma4.it (F.Q.); fabio.pigozzi@uniroma4.it (F.P.); 2 Department of Orthopaedic and Trauma Surgery, Campus Bio-Medico University of Rome, 00128 Rome, Italy; s.vasta@unicampus.it (S.V.); r.papalia@unicampus.it (R.P.)

**Keywords:** mental health, sports participation, injury, depression, anxiety

## Abstract

Sport participation and exercise practice are considered to be beneficial for mental status, yielding an improved mood and better quality of life. On the other hand, good mental fitness is thought to lead to better physical status and sport performance. The key aim of this narrative review is to provide an unstructured overview on the topic with special considerations on the role of mental and physical health to summarize the evidence on their reciprocal influence. While very few papers describe the role of mental health measures in affecting physical performance, more evidence is available concerning the effect of exercise and sport in improving mental health outcomes. Furthermore, there is a consistent role of depressive and anxiety symptoms in influencing the risk of sport injury in either recreational or elite athletes. Moreover, the importance of investigating mental health issues in elite and retired athletes is highlighted. On the basis of the available literature, several biases were found to affect the provided evidence mainly because of the complexity of a proper study design in this field.

## 1. Introduction

A key relationship between mental and physical health has been hypothesized and observed since ancient times with the Latin motto “mens sana in corpore sano” (healthy mind in a healthy body). The scientific literature provides several proofs of concept about this hypothesis, with many studies assessing the role of mental health on physical performance, and the role of physical fitness on mental performance and health [1,2]. Improving physical fitness constitutes a strategy to address the impact of an unhealthy lifestyle on mental health [3], with a specific impact on adolescents and young adults. The prevention of mental disturbances during this critical life period is based on the promotion of recreational and competitive sport participation. Indeed, involvement in sport activity is effective in reducing stress, depressive symptoms, general and social anxiety, and loneliness [4,5,6,7]. Similarly, for women, physical exercise may be effective in improving adverse physical, mental, and psychological changes experienced during the perimenopausal period [1]. Adolescents and perimenopausal women represent two examples of specific populations that are different in several biological and anthropological aspects for which physical activity provides a clear benefit. However, the drawbacks of excessive training or competitive sport activity on mental health increasing the risks of injury [8] and sport dropout [7] were reported. There is a clear distinction between sport participation and physical exercise. In some papers concerning measures of overall mental status [7] in adolescents, physical activity was defined as participation in recreational or competitive sport. In other studies investigating depressive symptoms and general wellbeing [9,10], cardiopulmonary fitness was used as a measure of physical fitness.

Thus, the non-standardized study design among the different papers led to difficulties in the interpretation of key results when comparing the evidence. In addition, several papers assessing the role of physical fitness on mental health were based on a correlational study design [11,12] often using univariate or small multivariate models, showing a direct association of physical activity or fitness indicators, and a plethora of psychological features and measures. However, correlational studies, rather than the causal effect of one or more variables on another, demonstrate the interdependency of several aspects concerning physical and psychological outcome measures. Thus, the hypothesis of a multidirectional influence of activity, exercise, sport participation, and mental health [13] seems to be more appropriate in the context of multidisciplinary medicine, where all aspects of people’s lifestyle contribute to the definition of their wellbeing and health.

Given the complex relationship of physical and psychological health, and the broad literature on the topic, the main aim of the present narrative review is to provide an unstructured overview of several papers available in the literature, compiling them into four major categories: (1) assessing the effect of mental health on metrics of physical performance; (2) assessing the effect of physical activity on metrics of mental health; and (3) assessing the effect of mental health indicators on the risk of injury during physical activities; (4) describing principal mental health issues in elite and retired athletes.

## 2. Materials and Methods

The topic of interest of the narrative review was the effect of sport and exercise participation on measures of mental fitness, and the effect of mental fitness on physical or sport performance metrics. Papers concerning the topic of interest were searched through the PubMed-Medline, Google Scholar, and Scopus online databases. Search queries were a combination of the following keywords: “mental fitness”, “mental health”, “sport”, “exercise”, “sport participation”, “physical fitness”.

All studies had to be longitudinal or cross-sectional clinical studies, including prospective and retrospective cohort studies and case series, and randomized trials. Furthermore, surveys and qualitative research papers (including reviews of the literature, guidelines, and consensus statements) were considered for inclusion if the results matched the aims of the review. We excluded case reports, duplicate outcomes of the same cohorts, children cohorts, and animal and laboratory studies. The publication date was not a restriction criterion for considering a paper in the review.

The papers were first screened by title and abstract to identify potentially relevant studies for the aim of the review. The full text of papers of potential interest was retrieved and fully read by two independent reviewers (G.T. and C.F.). If both reviewers agreed on the relevance of the manuscript, it was included for review. If disagreement could not be resolved through discussion, a third reviewer (S.V., A.G., or F.Q.) was asked to report on the manuscript inclusion.

Mental fitness was considered as a status of absence of symptoms related to psychological disturbances. Studies were considered for inclusion if results concerned outcomes of anxiety, depression, and mood and eating disturbances. Furthermore, the incidence or prevalence of symptomatic episodes for mental disturbances was considered of interest. Physical fitness was considered as active and regular participation in collective or individual sport or exercise activities, and performance in exercise and motor tasks. Physical or sport performance measures were considered if these dealt with sport or exercise participation, sport-related injuries, and measures of motor activity (see Table 1 for details).

Results of included studies were analyzed, and all manuscripts were divided according to the aims of the review: (1) studies concerning the effect of mental health on metrics of physical performance; (2) studies concerning the effect of physical exercise on mental health metrics; (3) studies concerning the role of mental health on the risk of injury. Furthermore, given the specific population, studies considering elite athletes cohorts or retired elite athletes were grouped together. A summary of the results of the included papers was provided, accordingly with the aforementioned categories.

## 3. Results

After the selection of retrieved studies, a total of 55 papers were included and analyzed to investigate relevant information concerning the assessment of (1) the effect of mental health on metrics of physical performance; (2) the effect of physical activity on metrics of mental health; and (3) the effect of mental health indicators on the risk of injury during physical activities. As elite athletes represent a very specific group of patients, results of papers dealing with these populations were separately reported.

### 3.1. Effect of Mental Health on Physical Performance Metrics

Concerning studies investigating the effects of psychological factors on sport performance, most focused on mindfulness-based intervention to improve sport performance and decrease injury rate [14,15,16]. The overall prevalence of mental disorders in sport populations was assessed in a survey-based study [15] that was performed on a cohort of 333 athletes, of which 22.8% were team athletes and 77.2% were individual athletes practicing 13 different sports (athletics, cross-country skiing, handball, canoe, orienteering, alpine skiing, gymnastics, basketball, equestrian, power lifting, swimming, golf, and sailing). This study found a general prevalence of mental health problems of 11.7% (females: 13.8%, males: 8.8%; *p* = 0.22), whereas the lifetime prevalence was 51.7% (females: 58.2%, males: 42.3%; *p* = 0.006). The onset of the first episode of mental symptoms peaked at age 19 with 50% of all onsets occurring at ages 17–21. Depressive disorders, eating disorders, trauma occurrence, and stress-related disorders were reported to be the most frequent. However, ADHD symptoms were also present in 5.4% (females: 5.1%, males: 5.8%; *p* = 0.96) of the athletes [15].

Mental fatigue is a psychobiological state caused by prolonged periods of demanding cognitive activity, and is characterized by a combination of specific subjective, behavioral, and physiological factors. A recent systematic review observed the effect of mental fatigue on physical performance leading to the conclusion that the duration and intensity of a physical task are fundamental in determining the decrease in physical performance due to mental fatigue [16]. Moreover, the most cognitively demanding sport and exercise tasks were the most mentally fatiguing when prolonged over time [17,18,19]. Furthermore, the endurance of moderately trained subjects was found to be reduced according to the reported mental fatigue. This effect reached a decrease of up to 15% in time to exhaustion for a fixed resistance cycling task [20], and a 2% to 5% increase in the completion time of a running task [18,21].

### 3.2. Effect of Physical Activity on Mental Health Metrics

Several studies investigated the effect that sport participation has on the development of a healthy mental status in adolescents [4,7,10,22,23,24,25]. In a longitudinal correlational study [7], the most relevant finding was the direct positive correlation between involvement in sport activities during childhood and mental fitness during adolescence. Stronger association was found for a minimum of 4 years of recreational sport participation (beta = 10.9; 95% confidence interval (CI): 2.60, 17.98) or for competitive sport involvement (beta = 19.48, 95% CI: 9.50, 29.46) with better mental health in late adolescence, measured through the Mental Health Continuum-Short Form (MHC-SF) [7]. A recent study on adolescents with a mean age of 12.5 years showed similar results, measuring motor competence through the PE Metrics™ (SHAPE America, Reston, Virginia, USA) and depressive symptoms through the Center for Epidemiologic Studies Depression Scale (CES-D). Correlation analysis showed an inverse association between cardiorespiratory fitness and depression score (beta = −0.24, *p* < 0.01) after correction for socioeconomic status [9]. Another study [22] among adolescents of the Nord-Trøndelag Health Study population measured psychological distress by assessing the Hopkins Symptom Check List Five items and self-esteem using a short version of the Rosenberg Self-Esteem Scale. According to the results, a high level of PA (more than 4 days per week) protected from psychological distress when compared to low level of PA (odds ratio 0.63, 95% CI: 0.46, 0.86 in females; OR 0.46, 95% CI: 0.27, 0.79 in males). A higher self-esteem score was significantly associated with team sport participation in females (OR 0.45, 95% CI 0.32, 0.64), and individual sport participation in males (OR 0.37, 95% CI 0.18 to 0.76) [22]. A paper by Jewett et al. [4] showed that sport participation during high school influences the mental status in adulthood. A significant association was reported between sport involvement during adolescence and lower depressive symptoms (F = 19.87, *p* < 0.001), lower perceived stress (F = 14.74, *p* < 0.001), and higher self-rated mental health (F = 14.65, *p* < 0.001) in early adulthood [4]. Stratification by activity level can predict health-related quality-of-life measures (Veterans RAND 12 Item Health Survey, VR-12) in collegiate athletes. The Mental Component Score of the VR-12 was positively associated with sport participation in general and activity level (*p* < 0.001) [23].

Within the adult participants of the Aerobics Center Longitudinal Study (ACLS), an ongoing NIH-funded study, an interim study on the collected data showed that depressive symptoms (CES-D) and emotional wellbeing (General Wellbeing Schedule; GWB) were significantly associated with maximal cardiorespiratory (CR) performance. In detail, inverse association was reported between CR performance and CES-D score in men and women (F = 28.45 and F = 13.27, respectively; *p* < 0.0001); similarly, strong positive association was found between CR fitness and GWB score in both men and women (F = 97.09 and F = 28.41, respectively; *p* < 0.0001). Moreover, physical activity level was positively correlated with an increase in GWB score (F = 78.7 in men and F = 24.82 in women; for both, *p* < 0.0001) [10]. In a cross-sectional correlational study [24] further evaluating CR fitness, the measurement of the Connor–Davidson Resilience Scale (CD-RISC) was used as a mediator in multivariable analysis, where positive correlation between CR and Short Form 12 quality of life was significant (*p* = 0.004). In this multivariable model, the mediation of resilience accounted for the 33.8% of the total correlational effect (*p* = 0.018) [24]. The effect of an 8-week program of physical activity (3 to 5 days per week, including football, boxing, and endurance training) was investigated on a population of refugees with symptoms of post-traumatic stress disorder. Participation to the program was inversely associated with self-reported anxiety symptoms (beta = –0.27, *p* < 0.05), and directly associated to self-perceived fitness (beta = 0.32, *p* < 0.01) and Health-Related Quality of Life outcome measure (beta = 0.32, *p* < 0.05) [25].

The effect of physical exercise on neuropsychiatric symptoms (BPSD) of dementia patients has also been investigated by several studies. A randomized trial on subjects affected by dementia compared the effect of a 12-week program of intense strengthening and balance exercises. Comparison with normal leisure activities showed higher improvement in the Bergs Balance Scale (2.9 vs. 1.2 points, *p* = 0.02); similarly, apathic symptoms were lower in those trained than those in the controls (*p* = 0.048) [26]. In addition to cognitive decline, BPSD very often occur in patients suffering from dementia with a prevalence ranging from 10% to 73% [27]. They are frequently very difficult to manage by pharmacological therapy, and extremely distressing for patients, and their families and caregivers. Several intervention studies investigating nonpharmacological therapeutic strategies for BPSD have focused on physical exercise. Although these studies are very heterogeneous in terms of the type and severity of dementia, and type and duration of the exercise intervention, they globally highlight a positive effect of physical exercise on BPSD [28,29,30,31]. Stella et al. [28] evidenced that a combination of aerobic and functional balance exercises 3 times a week for 6 months was able to reduce BPSD (agitation or aggression, depression, anxiety, apathy or indifference, irritability, and appetite alterations) as assessed by the Neuropsychiatric Inventory (NPI) in Alzheimer’s disease patients. Landi et al. [29] similarly reported the results of a pilot longitudinal study in which a moderate-intensity exercise program (combination of aerobic or endurance activities, strength training, balance, and flexibility training) reduced BPSD (wandering, physical and verbal abuse, and sleep disturbances) in frail nursing-home elderly patients with dementia. Neville et al. [30] showed that dementia-specific aquatic exercises designed by an exercise physiologist for strength, agility, flexibility, balance, and relaxation (twice a week for 12 weeks) in nursing-home residents with dementia reduced the number of BPSD as identified by the Revised Memory and Behavior Problems Checklist (RMBPC). An intervention study by Sampaio et al. [31] on 64 institutionalized older adults with dementia (38 patients in a 6-month supervised multicomponent exercise intervention group and 26 controls) showed a significant decrease in total BPSD score measured by NPI in the exercise group after 6 months of exercise. The performed intervention followed the recommendations of the American College of Sports Medicine, and consisted in aerobic, muscle-strengthening, flexibility, balance, and postural exercises with 2 sessions per week lasting 45–55 min [31]. Christofoletti et al. performed an observational study on 59 patients with dementia (AD, vascular, or mixed dementia), dividing them in three groups according to levels of physical activity that had been assessed by Modified Baecke Questionnaire for the Elderly (MBQE), data from a diary, pedometer, and an interview to caregivers and family members. BPSD were assessed by NPI. Results showed that patients with AD or vascular dementia who engaged in physical activity had fewer neuropsychiatric symptoms than those who did not [32].

A review by Thuné-Boyle et al. analyzed the scientific literature reporting the findings of the efficacy of physical activity on BPSD, showing that exercise could be beneficial in reducing some BPSD, especially depressed mood, agitation, and wandering, and may improve night-time sleep [33].

On the other hand, a systematic review by Forbes et al. found no clear evidence of the positive effects of exercise intervention on BPSD [34].

Besides the controversy, all studies agree that further work is needed to comprehend the potential of exercise as nonpharmacological therapy to manage BPSD in dementia patients.

There is some evidence in the scientific literature about the effect of physical exercise in psychiatric patients. Some observational studies evidenced that regular aerobic exercise is able to reduce the risk of developing major depressive disorders [35,36]. In patients with depression, performing regular exercise was able to reduce the frequency of hospitalization [36]. Moreover, patients with depression benefit through aerobic exercise on pharmacological therapy both in terms of speed of initial therapeutic response [37] and remission rates [38]. Differently from data obtained from aerobic training, trials highlighting the beneficial role of resistance training are few [39,40].

Physical activity is a protective factor against incident psychosis in the general population [41] and young people at high risk for psychosis [42]. Moreover, exercise represents a promising new treatment option, in addition to drugs and psychosocial interventions, for psychosis. There is some evidence supporting that exercise interventions (yoga and light stretching, moderately intense walking, bike riding, or team sports) may be beneficial for improving both positive and negative symptomatology, cognition, and quality of life in schizophrenia [43]. Recent studies suggest that exercise may also be useful in reducing antipsychotic medication side effects such as weight gain, and this action may help in improving compliance to pharmacological therapy for psychosis [44,45].

Some papers have discussed the relationship between sports injuries and mental health outcomes. A population study assessed the Youth Risk Behavior Surveillance Survey (YRBSS) among 5336 high school students. Of these, 3427 students were physically active, and 19.5% of these active subjects reported a concussion during the past 12 months. After controlling for confounding factors, students who had had a concussion had higher odds of self-harm (OR = 1.59, *p* = 0.003), depressive symptoms (aOR = 1.48, *p* = 0.006), attempted suicide (aOR = 3.10, *p* < 0.001), and injury from attempted suicide (aOR = 2.61, *p* = 0.006) [46]. Similar results were obtained in a study on retired football players, showing that a 9-year estimated risk of depression proportionally increased with the number of self-reported concussions (3% in those with no concussion episodes, 26.8% in those with 10 or more episodes (linear trend: *p* < 0.001) [47].

Anxiety spectrum symptoms were investigated through the Hospital Depression and Anxiety Scale (HADS) in retired rugby players who had reported concussion episodes. Results showed no significant difference in HADS and cognitive tests between concussed players and healthy controls, although concussion symptoms assessed through the Rivermead Post-Concussion Symptoms Questionnaire were significantly more persistent in those athletes reporting more than 9 episodes [48].

Recent systematic reviews on the mental outcomes in elite athletes who had suffered from concussion concluded that much evidence supports the effect of concussion in developing depressive symptoms, especially after retirement [49].

Conversely, there is insufficient evidence to suggest a positive relationship between concussion history and other long-term mental health outcomes such as anxiety, hostility, irritability, and aggression.

There is a dearth of studies that evaluate the relationship between subconcussive impact and mental-health measures, potentially due to the difficulty in quantifying these events [50].

### 3.3. Role of Mental Health in Injury during Sport Participation

Mental fitness can be considered as a prerequisite for healthy participation in sport, preventing from the possibility of overtraining, sport violence, and injuries. The mental conditions of an athlete approaching the sport season can be fundamental for a good performance and for avoiding injuries. In a report from 958 NCAA Division I athletes [51], depressive and anxiety symptoms were investigated before each season from 2007 and 2011. The 28.8% of the cohort reported anxiety symptoms (State-Trait Anxiety Inventory, STAI), and 21.7% reported depressive symptoms (CES-D). Those athletes reporting anxiety symptoms had a higher risk of injury (Relative Risk 2.3, 95% CI: 2–2.6, *p* < 0.001), while no increased risk was observed for those reporting depressive symptoms (RR 1.1, 95%CI: 0.9–1.3). Another report on elite athletes investigated the role for subjective wellbeing in the risk of injury during the season [52]. The Oslo Sports Trauma Research Centre (OSTRC) Overuse Injury Questionnaire was used for either monitoring injuries or assessing the wellbeing of the athlete. Males reported a significantly higher wellbeing score compared to females (*p* = 0.027), and females reported a significantly higher prevalence of injuries and severe injuries (*p* < 0.001 for both), and a higher severity score (*p* < 0.001). Correlation analysis showed also a negative association between wellbeing score and severity score (r = −0.32, *p* < 0.001), with a mixed model suggesting the influence of sex (*p* = 0.019) and severity score (*p* < 0.001) on wellbeing. Furthermore, mixed models showed that wellbeing score measured at the week before injury predicted injury (logistic mixed, *p* = 0.036) and injury severity (linear mixed, *p* = 0.010) [52].

### 3.4. Mental Health in Elite Athletes and Retired Elite Athletes

Professional athletes are far more subject to psychosocial pressure and performance goals than recreational practitioners are. For this specific population, the literature provides insights concerning the high number of stressors and their categories that an elite athlete must face [13,53], highlighting the different stress sources that are not limited to the challenge of specific performance, but also to public contexts, transfers, contracts, and team and press meetings [54]. Furthermore, the influence of those stressors may not end at retirement, but could also follow the athlete after the end of a brilliant career [55]. The eventuality of early career termination, such as an injury event, even more dramatically impacts the psychology of the subject [55]. For these reasons, within the key findings of the American Medical Society for Sports Medicine (AMSSM) Position Statement of 2019, the creation of a preretirement plan for older athletes by the sport authority and team direction is encouraged with grade A evidence [56]. A specific clinical entity defining the physical and psychological overload of elite athletes is overtraining syndrome (OTS), where the subjects must face multiple stressors, maintaining perfect performance [57]. Burnout affects 10% of elite athletes as a direct outcome of OTS [58]. The AMSSM Statement suggests a personalized treatment of OTS for individual athletes on the basis of multiple factors influencing their lifestyle [56]. Considering the attention that professional athletes pay to their physical shape, the incidence of eating disorders is high, especially in males, if compared to general population [59], with 11% of young elite athletes with disordered eating, and 19% of them on dietary treatment. For female athletes, a specific syndrome is often reported as the Female Athlete Triad, which is the combination of low energy availability, menstrual dysfunction, and impaired bone mineral density [60]. The three inter-related conditions are caused by the hard training and consequent hormonal imbalance that females face when involved in high-performance sports. An eating disorder can be part of the syndrome, being responsible of low energy availability, but does not necessarily meet the criteria for diagnosing anorexia or bulimia [60]. However, several eating disorder behaviors can be retrieved.

## 4. Discussion

The literature presents a wide variety of studies investigating the close relationship of mental health and sport performance, providing several insights on the topic. Regrettably, the direct cause–effect mechanisms are difficult to understand. The effect of mental health metrics on physical performance has been evaluated more in a qualitative environment, with no inferential insights about how much an impaired mental state could harm physical fitness. Conversely, several quantitative outcomes are provided in studies concerning involvement in sport activity and the consequent benefits on psychological measures [7,22]. However, correlation models are affected by several biases. First, the lack of large samples and correlation model power analyses prevents from understanding the consistency of results. Furthermore, the only multivariable model was presented by Pozuelo-Carrascosa et al. [24], with most of the papers showing results of univariate or multiple univariate analyses. Another consistent bias is represented by the lack of considering the lag effect in evaluating correlations, as the effects of physical activity on mental health measures could be observed after some time and over time. Only one paper by Von Rosen and Heijne considered this factor [52], assessing whether subjective wellbeing in a given week was potentially correlated with the risk of injury in the following week. Moreover, given the large variety of outcome measures in psychological assessment and the heterogeneity of study designs, meta-analysis of outcome data for the available papers’ results was not possible.

From an overall perspective, the literature confirmed with large amounts of evidence that a close and multifaceted relationship exists between mental health and physical performance. To systematically review the available evidence, it is advisable to strongly select the type of population and intervention, with a focus on one or a few specific characteristics of patients, paying attention to the difference among definitions of physical activity. Available scientific results are helpful in designing further studies to investigate the role of the specific psychological aspects in athletes and their effects on sport activities, physical performance, and the risk of injury.

## 5. Conclusions

Available scientific results are helpful in designing further studies to investigate the role of specific psychological aspects in athletes and their effects on sport activities, physical performance, and the risk of injury.

Moreover, regarding competitive and noncompetitive athletes, awareness of these sensitive issues may increasingly form part of the training of support personnel in order to guarantee the best physical and mental care to optimize athletic performance while protecting health.

## Figures and Tables

**Table 1:** Details of outcome measures in studies considered for review.

Mental Fitness Outcome Measures	Physical Fitness Outcome Measures
Overall or lifetime prevalence of mental health problems (depressive, eating, anxiety disorders, behavioral disturbances)	Participation in team or individual sport practice
Mental fatigue incidence or prevalence	Participation in training and exercise programs
General mental status questionnaires (MHC-SF, HSCL, GWB)	Sport-related injury (including concussion)
Depressive symptoms (CES-D, RSES)	Physical-task-based measures (time to exhaustion, time to complete)
Anxiety symptoms (HADS, STAI)	Cardiorespiratory performance
Neuropsychiatric symptoms (NPI)	Balance (Bergs Balance Scale)

CES-D: Center for Epidemiologic Studies Depression Scale, RSES: Rosenberg Self-Esteem Scale, MHC-SF: Mental Health Continuum-Short Form, HSCL: Hopkins Symptom Check List. GWB: General Wellbeing Schedule, HADS: Hospital Depression and Anxiety Scale, STAI: State-Trait Anxiety Inventory. NPI: Neuro-Psychiatric Inventory.
